# Circulating tumor cells and drug history in primary breast cancer patients

**DOI:** 10.20517/cdr.2019.79

**Published:** 2020-03-19

**Authors:** Silvia Jurisova, Marián Karaba, Gabriel Minarik, Juraj Benca, Tatiana Sedlackova, Daniela Manasova, Katarina Kalavska, Daniel Pindak, Jozef Mardiak, Michal Mego

**Affiliations:** ^1^2nd Department of Oncology, Faculty of Medicine, Comenius University, Bratislava 83310, Slovakia.; ^2^National Cancer Institute, Bratislava 83310, Slovakia.; ^3^Slovak Medical University, Bratislava 83101, Slovakia.; ^4^Institute of Molecular Biomedicine, Faculty of Medicine, Comenius University, Bratislava 83172, Slovakia.; ^5^Department of Medicine, St. Elizabeth University, Bratislava 81250, Slovakia.

**Keywords:** Circulating tumor cells, chronic medication, epithelial-to-mesenchymal transition, early breast cancer

## Abstract

**Aim:** Different types of chronic medication may affect breast cancer prognosis. Circulating tumor cells (CTCs) play an important role in cancer metastasis formation. There is no evidence of how chronic medication affects CTCs and breast cancer prognosis. The aim of this study was to evaluate association between chronic medication and CTCs in patients with primary breast cancer.

**Methods:** This study involved 414 patients with stage I-III primary breast cancer. Chronic drug history was collected from patients’ medical records and included all drugs that were prescribed for patients over at least the last 6 months prior to CTCs evaluation. CTCs were detected using a quantitative real-time polymerase chain reaction (qRT-PCR)-based method at the time of breast surgery.

**Results:** There was no association between CTCs, including their different subpopulations and chronic medication. Chronic medication using angiotensin-converting-enzyme inhibitors (ACEi), metformin, and insulin were associated with inferior disease-free survival (HR = 0.49, 95%CI 0.26-0.94, *P* = 0.007 for ACEi; HR = 0.27, 95%CI 0.08-0.91, *P* < 0.001 for metformin; and HR = 0.12, 95%CI 0.01-2.91, *P* < 0.001 for insulin) and this was most pronounced in patients with epithelial to mesenchymal transition (CTC_EMT) phenotype. In multivariate analysis, chronic administration of metformin and/or insulin was an independent predictor of inferior outcome.

**Conclusion:** Our findings show that there was no association between chronically used medication and CTCs in primary breast cancer patients. However, administration of ACEi, metformin, and/or insulin could negatively affect prognosis of patients with CTC_EMT.

## Introduction

According to global cancer statistics, breast cancer is the most commonly diagnosed cancer and the leading cause of cancer death among females, accounting for 23% of total cancer cases and 14% of cancer deaths^[1]^.

Circulating tumor cells (CTCs) are cancer cells intravasated into the blood stream after their separation from the primary tumor, which directly contribute to metastasis development^[2,3]^ and are also established as an independent predictor of progression-free and overall survival in patients with primary and metastatic breast cancer^[4-7]^. Although the connection between CTCs and bad prognosis is well described in breast cancer, CTCs are detected only in a subset of patients.

Comorbidities such as obesity, diabetes mellitus, hypertension, alcohol consumption, and non-cancer related drug exposure as well as regular physical activity may affect outcomes of breast cancer. Body mass index (BMI) ≥ 30 kg/m^2^ is a known factor responsible for increase in overall morbidity and mortality and is associated with breast cancer risk especially in postmenopausal women^[8-10]^.

Chronically used medications including non-steroidal anti-inflammatory drugs (NSAIDs), metformin, statins, and insulin may influence the progression of cancer^[11-14]^. However, there is limited evidence about how chronic medication can affect CTCs. In a previous study, it was shown that patients who were treated with statins before the diagnosis of inflammatory breast cancer (IBC) had significantly lower baseline CTC counts than patients not taking statins^[15]^. This observation was most pronounced in patients taking H-statins and was associated with improved progression-free and overall survival compared with non-statin users^[15]^. This could be attributed to the fact that certain types of statin may block a step involved in metastasis formation, including invasion, extravasation, epithelial-mesenchymal transition, and angiogenesis, and therefore may block pathways associated with cancer stem cells^[16]^. However, while clinical data support this observation in IBC, there are no data about the association between CTCs and statin use in non-IBC patients^[16]^.

In this study, we hypothesized that certain types of chronic medication utilized before diagnosis of primary breast cancer could correlate with presence of CTCs in peripheral blood. The aim of our study was to evaluate an association between different CTC subpopulations and chronic medication and/or whether these drugs could be linked to patients’ outcomes in primary breast cancer.

## Methods

### Study patients

This was a prospective translational study that evaluated prognostic value of CTCs in stage I-III primary breast cancer, including 427 patients, as described previously^[17]^. For this sub-study, 414 patients for whom complete medical history, including drug history, was available were eligible. Chronic drug history was collected from the medical records and included all drugs that were prescribed for patient over at least the last six months before date of surgery, when CTCs were evaluated. Chronic medication was categorized into several classes including NSAIDs, *L*-thyroxin, angiotensin-converting-enzyme inhibitors (ACEi), sartans, anticoagulants (low molecular weight heparin and/or warfarin), betablockers, statins, metformin, and insulin. BMI was calculated at the time of surgery.

All study participants provided signed informed consent before study enrollment. The study was approved by the Institutional Review Board (IRB) of the National Cancer Institute of Slovakia and was conducted between March 2012 and February 2015. Healthy donors (*n* = 60) were age-matched women without breast cancer who were enrolled according to the IRB-approved protocol and all of them signed informed consent, as described previously^[17]^.

### Detection of CTC in peripheral blood

Quantitative real-time polymerase chain reaction (qRT-PCR) assay was used for CTCs detection in peripheral blood that was previously depleted of CD45+ cells for CTCs enrichment, as described previously^[17-20]^.

### CTC definition

Patient samples with higher *KRT19* gene transcripts than those of healthy donors were scored as epithelial CTCs positive (CTC_EP), while patient samples with higher Epithelial-mesenchymal transition transcription factor (*TWIST1, SNAIL1, SLUG*, and *ZEB1*) gene transcripts than those of healthy donors were scored as CTC_EMT positive. Expression of at least one of the markers (either epithelial or mesenchymal) at levels above the defined cut-off was sufficient to define a sample as CTC positive^[18,20]^.

### Statistical analysis

The patients’ characteristics were summarized using the median (range) for continuous variables and frequency (percentage) for categorical variables. The median follow-up period was calculated as a median observation time among all patients and among those still alive at the time of their last follow-up. Disease-free survival (DFS) was calculated from the date of CTC measurement to the date of disease recurrence (locoregional or distant), secondary cancer, death, or last follow-up. DFS was estimated using Kaplan-Meier product limit method and compared between groups by log-rank test. Cox-Mantel hazard ratio and 95%CI for Cox-Mantel hazard ratio were calculated as well. Univariate analyses with Chi squared or by the Fisher’s exact test were performed to find association between drug history and CTC status.

A multivariate Cox proportional hazards model for DFS was used to assess differences in outcome on the basis chronic medication, CTC_EMT status (present *vs.* absent), hormone receptor status (positive for either *vs.* negative for both), HER-2 status (positive or negative), tumor size (T1 *vs.* T2 and higher), axillary lymph node involvement (N0 *vs.* N+), and Ki67 status (< 20% *vs.* > 20%). Step-wise regression techniques were used to build multivariate models using a significance level of 0.10 to remain in the model. All *P* values presented are two-sided, and associations were considered significant if the *P* value is less than or equal to 0.05. Statistical analyses were performed using NCSS 11 Statistical Software (2016). NCSS, LLC. Kaysville, Utah, USA, ncss.com/software/ncss.

## Results

Overall, 414 patients with primary breast cancer were included in this analysis. Patients’ characteristics are shown in Table 1. Median age of patients in this cohort was 60 years (range: 25-83 years). The majority of patients were of good prognosis, with tumor size less than 2 cm (69.3%), without axillary lymph nodes involvement (65.0%), and with low/intermediate grade (65.5%). CTC_EP was detected in 48 patients (11.6%), while CTC_EMT in 73 patients (17.6%); any type of CTC was present in 113 patients (27.3%).

**Table 1:** Patients’ characteristics

	*n*	%
All patients	414	100.0
T-stage		
T1	287	69.3
> T1	127	30.7
Histology		
IDC	352	85.0
Other	62	15.0
Grade		
Low and intermediate	271	65.5
High grade	132	31.9
Unknown	11	2.7
Lymph nodes		
N0	269	65.0
N+	140	33.8
Unknown	5	1.2
Hormone receptor status (cut-off 1%)		
Negative for both	354	85.5
Positive for either	60	14.5
HER2 status		
Negative	352	85.0
Positive	62	15.0
Ki67 status (cut-off 20%)		
< 20%	249	60.1
> 20%	163	39.4
Unknown	2	0.5
Molecular subtype		
Luminal A	211	51.0
Luminal B	97	23.4
HER2+	62	15.0
TN	42	10.1
Unknown	2	0.5
CTC_EP	48	11.6
CTC_EMT	73	17.6
CTC_Any	113	27.3

IDC: invasive ductal carcinoma; TN: triple negative; HER2: receptor tyrosine-protein kinase erbB-2; CTC_EP: circulating tumor cell with epithelial phenotype; CTC_EMT: circulating tumor cell with epithelial-mesenchymal transition phenotype

### Association between chronic medication and CTC status

Associations between CTC and chronic medication are shown in Tables 2-4. There was no association between CTC, including different subpopulations and chronic medication, except the trend for association between CTC_EP and ACEi/sartans, where patients on ACEi/sartans had lower prevalence of CTC_EP compared to no ACEi/sartan (7.9% *vs.* 16.1%, *P* = 0.06). Association between BMI status and CTC was not detected [Table 5].

**Table 2:** Association between drug history and CTC_EP

Drug			CTC_EP negative		CTC_EP positive		*P*-value
		*n*	*n*	%	*n*	%	
NSAID	No	340	293	86.2	47	13.8	1.00
	Yes	9	8	88.9	1	11.1	
*L*-thyroxin	No	307	263	85.7	44	14.3	0.48
	Yes	42	38	90.5	4	9.5	
ACEi	No	290	246	84.8	44	15.2	0.10
	Yes	59	55	93.2	4	6.8	
Sartans	No	305	261	85.6	44	14.4	0.48
	Yes	44	40	90.9	4	9.1	
ACEi/Sartan	No	248	208	83.9	40	16.1	0.06
	Yes	101	93	92.1	8	7.9	
Betablockers	No	250	218	87.2	32	12.8	0.40
	Yes	99	83	83.8	16	16.2	
Statins	No	295	251	85.1	44	14.9	0.20
	Yes	54	50	92.6	4	7.4	
Metformin	No	332	285	85.8	47	14.2	0.49
	Yes	17	16	94.1	1	5.9	
Insulin	No	345	297	86.1	48	13.9	1.00
	Yes	4	4	100.0	0	0.0	
Insulin/Metformin	No	329	282	85.7	47	14.3	0.33
	Yes	20	19	95.0	1	5.0	
LMWH/Warfarin	No	335	288	86.0	47	14.0	0.70
	Yes	14	13	92.9	1	7.1	

NSAID: non-steroidal anti-inflammatory drugs; ACEi: angiotensin-converting-enzyme inhibitors; LMWH: low molecular weight heparin; CTC_EP: circulating tumor cell with epithelial phenotype

**Table 3:** Association between drug history and CTC_EMT

Drug			CTC_EMT negative		CTC_EMT positive		*P*-value
		*n*	*n*	%	*n*	%	
NSAID	No	366	293	80.1	73	19.9	0.36
	Yes	8	8	100.0	0	0.0	
*L*-thyroxin	No	325	263	80.9	62	19.1	0.57
	Yes	49	38	77.6	11	22.4	
ACEi	No	306	246	80.4	60	19.6	1.00
	Yes	68	55	80.9	13	19.1	
Sartans	No	319	261	81.8	58	18.2	0.14
	Yes	55	40	72.7	15	27.3	
ACEi/Sartan	No	254	208	81.9	46	18.1	0.33
	Yes	120	93	77.5	27	22.5	
Betablockers	No	267	218	81.6	49	18.4	0.39
	Yes	107	83	77.6	24	22.4	
Statins	No	312	251	80.4	61	19.6	1.00
	Yes	62	50	80.6	12	19.4	
Metformin	No	352	285	81.0	67	19.0	0.40
	Yes	22	16	72.7	6	27.3	
Insulin	No	370	297	80.3	73	19.7	1.00
	Yes	4	4	100.0	0	0.0	
Insulin/Metformin	No	349	282	80.8	67	19.2	0.60
	Yes	25	19	76.0	6	24.0	
LMWH/Warfarin	No	358	288	80.4	70	19.6	1.00
	Yes	16	13	81.3	3	18.8	

NSAID: non-steroidal anti-inflammatory drugs; ACEi: angiotensin-converting-enzyme inhibitors; LMWH: low molecular weight heparin; CTC_EMT: circulating tumor cell with epithelial-mesenchymal transition phenotype

**Table 4:** Association between drug history and CTC

Drug			CTC_Any negative		CTC_Any positive		*P*-value
		*n*	*n*	%	*n*	%	
NSAID	No	405	293	72.3	112	27.7	0.45
	Yes	9	8	88.9	1	11.1	
*L*-thyroxin	No	362	263	72.7	99	27.3	1.00
	Yes	52	38	73.1	14	26.9	
ACEi	No	343	246	71.7	97	28.3	0.38
	Yes	71	55	77.5	16	22.5	
Sartans	No	355	261	73.5	94	26.5	0.35
	Yes	59	40	67.8	19	32.2	
ACEi/Sartan	No	287	208	72.5	79	27.5	0.91
	Yes	127	93	73.2	34	26.8	
Betablockers	No	296	218	73.6	78	26.4	0.54
	Yes	118	83	70.3	35	29.7	
Statins	No	350	251	71.7	99	28.3	0.36
	Yes	64	50	78.1	14	21.9	
Metformin	No	392	285	72.7	107	27.3	1.00
	Yes	22	16	72.7	6	27.3	
Insulin	No	410	297	72.4	113	27.6	0.58
	Yes	4	4	100.0	0	0.0	
Insulin/Metformin	No	389	282	72.5	107	27.5	0.82
	Yes	25	19	76.0	6	24.0	
LMWH/Warfarin	No	397	288	72.5	109	27.5	1.00
	Yes	17	13	76.5	4	23.5	

NSAID: non-steroidal anti-inflammatory drugs; ACEi: angiotensin-converting-enzyme inhibitors; LMWH: low molecular weight heparin; CTC: circulating tumor cell

**Table 5:** Association between BMI and CTC

BMI		CTC_EP negative		CTC_EP positive		*P*-value
	*n*	*n*	%	*n*	%	
< 25	180	157	87.2	23	12.8	0.47
26-30	118	107	90.7	11	9.3	
31-35	92	80	87.0	12	13.0	
> 36	23	22	95.7	1	4.3	
		**CTC_EMT negative**		**CTC_EMT positive**		***P*-value**
< 25	180	150	83.3	30	16.7	0.46
26-30	118	99	83.9	19	16.1	
31-35	92	75	81.5	17	18.5	
> 36	23	16	69.6	7	30.4	
		**CTC_Any negative**		**CTC_Any positive**		***P*-value**
< 25	180	132	73.3	48	26.7	0.60
26-30	118	90	76.3	28	23.7	
31-35	92	64	69.6	28	30.4	
> 36	23	15	65.2	8	34.8	

BMI: body mass index; CTC: circulating tumor cell; CTC_EP: circulating tumor cell with epithelial phenotype; CTC_EMT: circulating tumor cell with epithelial-mesenchymal transition phenotype

### Disease outcome according to chronic medication

**Figure 1:**
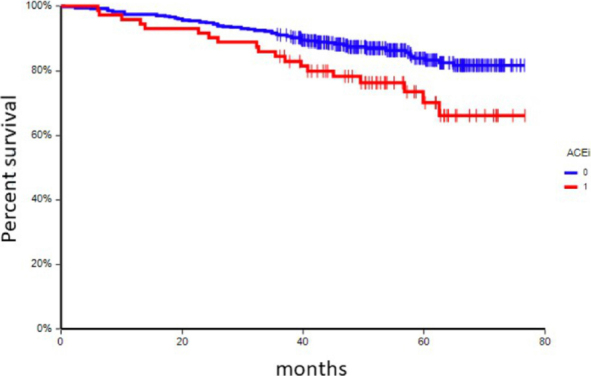
Kaplan-Meier estimates of probabilities of disease-free survival according to chronic administration of ACEi (*n* = 414), HR = 0.49, 95%CI 0.26-0.94, P = 0.007. HR: hazard ratio

**Figure 2:**
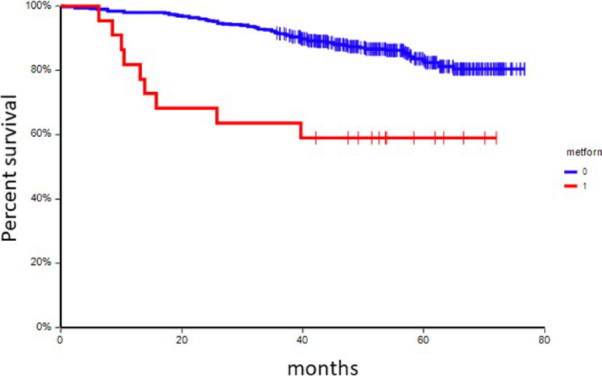
Kaplan-Meier estimates of probabilities of disease-free survival according to chronic administration of metformin (*n* = 414), HR = 0.27, 95%CI 0.08-0.91, *P* < 0.001. HR: hazard ratio

**Figure 3:**
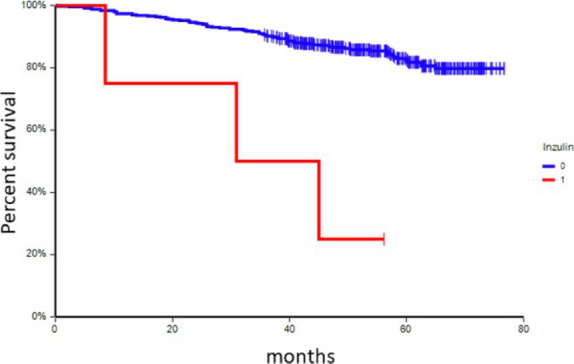
Kaplan-Meier estimates of probabilities of disease-free survival according to chronic administration of insulin (*n* = 427), HR = 0.12, 95%CI 0.01-2.91, *P* < 0.001. HR: hazard ratio

**Figure 4:**
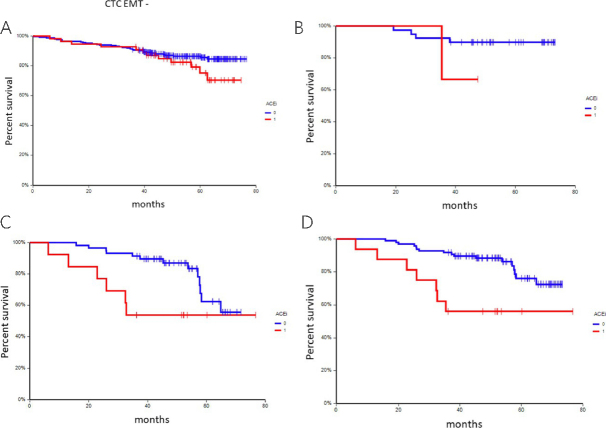
Kaplan-Meier estimates of probabilities of disease-free survival according to CTC status A, CTC negative B, CTC_EP positive C, CTC_EMT positive D, CTC_any. CTC: circulating tumor cell; CTC_EP: circulating tumor cell with epithelial phenotype; CTC_EMT: circulating tumor cell with epithelial-mesenchymal transition phenotype

At a median follow-up time of 55.0 months (range: 4.9-76.7 months), 74 patients (17.3%) had experienced a DFS event, and 36 patients (8.4%) had died. In univariate analysis, chronic administration of ACEi, metformin, and/or insulin was associated with inferior DFS [Table 6 and Figures 1-3] This correlation was most pronounced in patients with CTC_EMT phenotype. The negative prognostic impact of chronic medication was especially observed in patients with CTC_EMT that were on ACEi compared to patients with CTC_EP and/or no CTCs, where administration of ACEi had no impact on patient’s prognosis [Table 7 and Figure 4].

**Table 6:** Impact of drug history on disease-free survival in primary breast cancer

Drug	*n*	HR*	95% Low**	95% High**	*P*-value***
No NSAID NSAID	405 9	0.00	0.00	0.00	0.200
No *L*-thyroxin *L*-thyroxin	362 52	0.93	0.45	1.91	0.840
No ACEi ACEi	343 71	0.49	0.26	0.94	0.007
No sartans Sartans	355 59	0.69	0.35	1.37	0.230
No ACEi/sartan ACEi/sartan	287 127	0.53	0.31	0.89	0.008
No betablockers Betablockers	296 118	0.82	0.49	1.40	0.450
No statins Statins	350 64	0.63	0.32	1.22	0.110
No metformin Metformin	392 22	0.27	0.08	0.91	< 0.001
No insulin Insulin	410 4	0.12	0.01	2.91	< 0.001
No insulin/metformin Insulin/metformin	389 25	0.24	0.08	0.77	< 0.001
No LMWH/warfarin LMWH/warfarin	397 17	1.43	0.43	4.71	0.620

*Cox-Mantel hazard ratio; **95%CI for Cox-Mantel hazard ratio; ***equal-weighted logrank test. HR: hazard ratio; NSAID: non-steroidal anti-inflammatory drugs; ACEi: angiotensin-converting-enzyme inhibitors; LMWH: low molecular weight heparin

**Table 7:** Impact of drug history on disease-free survival in primary breast cancer according to CTC subgroups

	Sample size 0/1	HR*	95% Low**	95% High**	*P*-value***
No NSAID *vs.* NSAID					
CTC negative	293/8	0.00	0.00	0.00	0.253
CTC_EP positive	47/1	0.00	0.00	0.00	0.739
CTC_EMT positive	NA	NA	NA	NA	NA
CTC_any	112/1	0.00	0.00	0.00	0.673
No *L*-thyroxin *vs.* t-thyroxin					
CTC negative	263/38	0.89	0.36	2.18	0.782
CTC_EP positive	44/4	0.30	0.01	9.38	0.251
CTC_EMT positive	62/11	1.18	0.37	3.77	0.785
CTC_any	99/14	1.10	0.34	3.54	0.874
No ACEi *vs.* ACEi					
CTC negative	246/55	0.58	0.27	1.26	0.104
CTC_EP positive	44/4	0.36	0.01	8.7	0.339
CTC_EMT positive	60/13	0.36	0.1	1.31	0.029
CTC_any	97/16	0.27	0.07	1.03	0.002
No sartan *vs.* sartan					
CTC negative	261/40	0.67	0.28	1.62	0.308
CTC_EP positive	44/4	0.00	0.00	0.00	0.490
CTC_EMT positive	58/15	0.78	0.27	2.31	0.636
CTC_any	94/19	0.79	0.27	2.28	0.642
No ACEi/sartan *vs.* ACEi/sartan					
CTC negative	208/93	0.57	0.3	1.09	0.063
CTC_EP positive	40/8	0.82	0.08	8.44	0.856
CTC_EMT positive	46/27	0.50	0.20	1.27	0.114
CTC_any	79/34	0.43	0.18	1.07	0.036
No blockers *vs.* blockers					
CTC negative	218/83	0.93	0.48	1.79	0.817
CTC_EP positive	32/16	1.98	0.31	12.73	0.535
CTC_EMT positive	49/24	0.51	0.2	1.34	0.131
CTC_any	78/35	0.72	0.3	1.72	0.431
No statin *vs.* statin					
CTC negative	251/50	0.53	0.24	1.17	0.054
CTC_EP positive	44/4	0.00	0.00	0.00	0.490
CTC_EMT positive	61/12	0.92	0.26	3.26	0.892
CTC_any	99/14	0.85	0.23	3.07	0.787
No metformin *vs.* metformin					
CTC negative	285/16	0.37	0.09	1.53	0.027
CTC_EP positive	47/1	0.00	0.00	0.00	0.739
CTC_EMT positive	67/6	0.13	0.01	1.6	< 0.001
CTC_any	107/6	0.10	0.01	1.78	< 0.001
No Insulin *vs.* Insulin					
CTC negative	297/4	0.11	0.00	3.00	< 0.001
CTC_EP positive	NA	NA	NA	NA	NA
CTC_EMT positive	NA	NA	NA	NA	NA
CTC_any	NA	NA	NA	NA	NA
No Insulin/metformin *vs.* Insulin/metformin					
CTC negative	282/19	0.29	0.08	1.1	0.002
CTC_EP positive	47/1	0.00	0.00	0.00	0.739
CTC_EMT positive	67/6	0.13	0.01	1.6	< 0.001
CTC_any	107/6	0.1	0.01	1.78	< 0.001
No LMWH *vs.* LMWH					
CTC negative	288/13	0.96	0.23	4.08	0.960
CTC_EP positive	47/1	0.00	0.00	0.00	0.739
CTC_EMT positive	70/3	0.00	0.00	0.00	0.312
CTC_any	109/4	0.00	0.00	0.00	0.348

*Cox-Mantel hazard ratio; **95%CI for Cox-Mantel hazard ratio; ***equal-weighted logrank test. NSAID: non-steroidal anti-inflammatory drugs; ACEi: angiotensin-converting-enzyme inhibitors; LMWH: low molecular weight heparin; CTC: circulating tumor cell; CTC_EP: circulating tumor cell with epithelial phenotype; CTC_EMT: circulating tumor cell with epithelial-mesenchymal transition phenotype; HR: hazard ratio; NA: not applicable

In multivariate analysis, presence of CTC_EMT, axillary nodal involvement, hormone receptor status, Ki67 status, and chronic prescription of insulin/metformin were independently associated with DFS [Table 8].

**Table 8:** Multivariate analysis of factors associated with disease-free survival

Variable	HR	95% Low	95% High	*P*-value
N stage N+ *vs*. N0	2.43	1.50	3.94	0.0003
ER/PR status positive for either *vs.* negative for both	0.51	0.28	0.94	0.0315
Ki 67 > 20% *vs.* < 20%	2.33	1.36	4.00	0.0021
ACEi yes *vs.* no	1.71	0.94	3.08	0.0766
Insulin/metformin yes *vs.* no	3.97	1.95	8.08	0.0001
CTC_EMT present *vs.* absent	2.44	1.43	4.15	0.0010

HR: hazard ratio; ER: estrogen receptor; PR: progesteron receptor; CTC_EMT: circulating tumor cell with epithelial-mesenchymal transition phenotype; ACEi: angiotensin-converting-enzyme inhibitors

## Discussion

In this translational study, we observed no association between CTCs, including their different subpopulations and chronic medication, except the trend for association between CTC_EP and ACEi/sartans, where patients on ACEi/sartans had lower prevalence of CTC_EP compared to patients without ACEi/sartans. We did not noticed association between BMI status and CTCs as well. These data suggest that chronic medication for general co-morbid conditions have only a slight impact on metastatic cascade. Moreover, we observed that chronic administration of ACEi, metformin, and/or insulin was associated with inferior DFS, while, in multivariate analysis, only insulin/metformin remained independently associated with clinical prognosis. CTC status had no effect on patient’s outcome according to chronic medication; however, due to small size of several subgroups, e.g., NSAID, low molecular weight heparin, and others, the statistical power for analysis is limited.

Approximately 16% of breast cancer patients have diabetes^[21]^. Diabetes mellitus not only increases the risk of breast cancer, but might also worsen breast cancer prognosis^[21]^. Insulin resistance and hyperinsulinemia state may be a potential mediator of this effect^[22]^. In our study, chronic use of metformin and insulin was associated with inferior outcome. Previously, it was shown that continual administration of insulin over ≥ 3 years was associated with an increased risk of mortality in breast cancer^[23]^. Moreover, fasting hyperinsulinemia was reported to be an independent predictor for higher risk of breast cancer distant recurrence and death in women without known diabetes^[24]^. Insulin is known as an enhancer of cancer cell proliferation, inhibiting apoptosis by its receptor and insulin-like growth factor through the PI3K/Akt and MAPK pathways^[22]^. The other reason contributing to progression of breast cancer is decrease of plasma levels of sex hormone binding globulin related to insulin, which results in increase of endogenous estrogen and androgen levels^[22]^. Contrary to our study, metformin, front-line therapy for the treatment of type 2 diabetes, especially in overweight and obese patients, may reduce breast cancer incidence and improve prognosis by several potential mechanisms according to some preclinical data^[24,25]^. We suppose that, in our trial, worse prognosis associated to metformin and/or insulin administration could be related to diabetes as comorbid condition. Data related to glycemic control and insulinemia were not available. Therefore, we cannot exclude that poor glycemic control and/or hyperinsulinemia might influence this observation. The most negative impact on DFS was observed in CTC_EMT positive subtype. However, our data suggest that worse prognosis related to these drugs might not be related to more efficient metastatic cascade, as there were no differences between CTC and this antidiabetic medication. Due to limited sample size, however, we cannot exclude limited statistical power to definitively answer this question, while the multiple testing approach could affect study results as well. Therefore, our results are only hypothesis generating and validation studies are needed.

Certain classes of antihypertensive drugs are associated with shorter survival in several types of cancers. However, the connection between antihypertensive agents and cancer patient survival remains unclear^[26]^. ACEis/angiontensin receptor antagonists are the most active drugs approved for treatment of hypertension, heart failure, and diabetic nephropathy. Several epidemiological studies have investigated relationship between ACEi use and cancer-specific mortality in patients with breast cancer^[26-30]^. In some of them, there was a small increase in cancer recurrence with ACEi use, while others suggest that this drug could be safely administered to breast cancer patients, without affecting breast cancer outcome. In our study, chronic administration of ACEi was associated with inferior DFS, and this was most pronounced in patients with CTC_EMT phenotype. Contrary to this observation, there was no association between ACEi and CTC count.

In conclusion, our findings show that there was no association between chronically used medication and/or CTCs in patients with primary breast cancer, while chronic administration of ACEi, metformin, and insulin could negatively affect prognosis. These data suggest that evaluated chronic medications are not able to favorably affect biology of primary breast cancer.

## Declarations

### Availability of data and materials

All data source could be available to readers on request.

### Copyright

© The Author(s) 2020.
